# Supplementary material for: Are carbon emissions trading and green financial instruments synergistic? -Comprehensive quantitative research based on content analysis
Source: PLoS One. 2024 Mar 7;19(3):e0298601. doi: 10.1371/journal.pone.0298601 (PMC10919601; doi:10.1371/journal.pone.0298601)
Supplement: S1 Data — (ZIP) [file pone.0298601.s001.zip › Data/地级市Z_F工作报告.zip!/║╙─╧/┬σ╤⌠╩╨/└┤╫╘╣1⁄2╓┌║┼í╛┬φ┐╦╩2╛▌═°í┐_2011.docx]

各位代表：

现在，我代表市人民政府向大会作工作报告，请予审议，并请各位政协委员和列席人士提出意见。

一、团结奋进，我们顺利实现了五年经济总量翻一番的宏伟目标

2010年，是“十一五”的收官之年，也是我市经济形势较为复杂的一年。在省委、省政府和市委的正确领导下，在市人大、市政协的监督支持下，市政府紧紧依靠全市人民，科学应对，奋力攻坚，圆满完成了市十三届人大二次会议确定的各项目标任务。

初步测算，全年完成地区生产总值2321.2亿元，同比增长13.2%，分别高于全国、全省2.9和1个百分点。地方财政一般预算收入142亿元，增长18.1%。全社会固定资产投资1768.8亿元，增长22.2%、高于全省1.2个百分点；其中城镇投资1548.8亿元，增长21%。社会消费品零售总额808.8亿元，增长19%。外贸出口10.5亿美元，增长61.7%，分别高于全国、全省30.4和18.2个百分点，提前三个月超额完成省定目标。城镇居民人均可支配收入和农民人均纯收入分别达到17639元和5680元，高于全省1709元和157元。金融机构存贷款余额分别达到2096.1亿元和1113.9亿元，比年初增加376.3亿元和246.8亿元，增量均居全省第二位。

一年来，我们在保持经济平稳较快增长的同时，更加注重以项目为抓手，加快产业优化升级。运行协调更加有效。相继实施了促进经济平稳较快发展的十大行动计划以及工业运行、项目建设、招商引资三个百日行动计划，确保了经济平稳运行。全市规模以上工业实现增加值1014亿元，增长21.8%，增速分别高于全国、全省6.1和2.8个百分点，居全省第三位；实现利润189.4亿元，增长39.6%、高于全省3.7个百分点。工业结构不断优化。实施千万元以上工业项目2191个，完成投资844.3亿元。一拖工业园、新洛轴一期、四季沐歌太阳能基地一期等1724个项目竣工投产；龙鼎铝业60万吨高精度铝箔等467个项目加快实施；龙泉金亨电力2×60万千瓦机组、河南煤化集团90万吨PTA等20个项目前期工作顺利推进。装备制造、能源电力、石化、硅光电、新材料等五大优势产业增加值占规模以上工业的71.5%。高新技术产业增加值140亿元，增长27%。服务业发展步伐加快。实施千万元以上服务业项目129个，完成投资118亿元。正大城市广场等24个亿元以上项目加快推进。白马寺印度风格佛殿落成开放。白云山被评为国家 5A 级景区。第28届牡丹花会和河洛文化旅游节圆满成功。全年接待游客6079万人次，其中入境游45.8万人次、约占全省的三分之一，旅游总收入302亿元。房地产完成投资179.9亿元，增长30.4%。产业集聚区建设强力推进。17个产业集聚区共完成固定资产投资474.3亿元，其中基础设施投资114.7亿元。实现企业营业收入2446.9亿元，增长37.7%。完成税收102.4亿元，增长23.7%。

我们在全面推进“三农”工作的同时，更加注重农民增收和农村生产生活条件的改善。现代农业加快发展。粮食总产47.2亿斤，连续7年实现丰收。牡丹、核桃、蔬菜、烟叶、畜牧业等特色产业快速发展。新增龙头企业32家、农民专业合作社392家，产业化占农业总产值的比重达到57%，产业化经营占农户总收入的52%。转移农村富余劳动力142万人，农民务工收入占现金收入的40%。新农村建设不断加强。完成水库除险加固39座。建成通组通户道路3010公里。开工新型农村社区71个。户用沼气发展到50万座。植树造林44.5万亩，顺利通过国家森林城市验收。我市荣获第二届全国绿博会金奖，再夺省红旗渠精神杯。

我们在加快中心城市建设的同时，更加注重推进城乡一体化进程。新区建设全面提速。实施重大项目227个，完成投资205.7亿元，伊河综合治理、路网基础工程、中央商务区等重大项目加快实施，洛河市区段东西延伸治理开工建设。洛河以北城区改造加快推进。旧城及城中村改造完成投资96.5亿元，拆迁178.2万平方米，开工447.8万平方米，竣工296.9万平方米。华山北路一期等13项工程建成投用。交通基础设施建设进展顺利。郑卢高速、连霍高速洛阳段，洛栾高速，洛偃、洛吉、洛宜快速通道等项目加快建设。县城、小城镇建设成效显著。县城建设及城中村改造完成投资43亿元，拆迁210万平方米，开工430万平方米，竣工210万平方米。

我们在加快推进各项改革、增强内生动力的同时，更加注重提升开放水平。企事业单位改革稳步推进。河柴集团政策性破产全面完成，洛轴集团政策性破产基本完成，洛玻集团职工分流安置已近尾声。煤炭企业兼并重组工作大头落地。事业单位改革试点成效明显。其它领域改革步伐加快。市县政府机构改革顺利完成，集体林权制度改革超额完成任务，文化体制改革加快推进，公立医院改革试点正式启动。招商引资成果丰硕。全年实际利用外资12.1亿美元、市外境内资金330.7亿元，分别增长31.6%和38%。我市被评为全省对外开放工作先进单位。民营经济快速发展。民营企业新上投资500万元以上项目565个，总投资326亿元；新设担保机构30家，新增注册资本金13.5亿元、担保额55.5亿元。民营企业营业收入、增加值分别增长22%和26%。

我们在加快经济发展的同时，更加注重集中财力改善民生。省定、市定实事圆满完成。中州西路西延长线改造等10类66件省定实事、50件市定实事全部完成。全年各级财政对民生的投入达153亿元，占财政一般预算支出的66.7%。就业再就业工作扎实推进。新增城镇就业13.8万人，其中再就业5.4万人、困难人员再就业2.3万人。新开发公益岗位1200个，实现了零就业家庭至少有一人就业。社会事业繁荣发展。新区洛一高和龙康小区、滨河小区中学竣工投用。新区二外、龙泰中学、市第一中医院、精神卫生中心等项目进展顺利。建成乡镇综合文化站28个、村基层服务点1020个。社会保障水平不断提高。农村养老保险、失地农民社会保障工作稳步推进。城镇居民医疗保险和工伤保险实现市级统筹。4833户受灾群众冬季前全部入住新房。改造农村危房1.15万户，新建、改扩建农村敬老院27所。新开工廉租房35.3万平方米、经济适用房57.2万平方米，分别竣工29.3万平方米和34.7万平方米。

我们在加强民主法制和精神文明建设的同时，更加注重保持社会和谐稳定。依法行政水平不断提高。自觉接受人大的法律监督和政协的民主监督，办理人大代表建议233件、政协委员提案464件，办结率、满意率均为100%。出台地方性法规2部、政府规章4件，集中清理规范性文件526件。深入开展“两转两提”活动，廉政建设不断加强，机关效能明显提高。文明创建活动蓬勃开展。积极创建全国双拥模范城六连冠，驻洛部队和民兵预备役为地方发展做出了重要贡献。创建全国文明城市工作扎实推进。我市第七次蝉联全国无偿献血先进城市称号。平安建设成绩突出。警务机制改革稳步推进，技防全覆盖工程全部完成，打黑除恶扫痞行动取得阶段性成果。完善领导干部接访等制度，解决了一大批群众反映的热点难点问题。创新便民服务方式，110城市应急平台初步建立。我市再获全省平安建设先进市称号。统计、外事、侨务、地震、气象、民族宗教、档案、地方史志、人口和计划生育、残疾人事业等都取得了新的成绩。

各位代表！五年前，市十二届人大三次会议提出了“十一五”末实现全市经济总量翻一番的宏伟目标。经过五年的攻坚，特别是2010年的奋力拼搏，“十一五”规划的宏伟目标圆满完成，实现了洛阳经济社会发展的又一次跨越。

五年快速发展，经济总量实现翻番。“十一五”期间，全市生产总值由1000亿元迈上2000亿元台阶，年均增长14.6%，不仅高于“十五”期间1.1个百分点，而且分别高于全国、全省3.4和1.8个百分点；人均生产总值由17486元增加到35000元以上；地方财政一般预算收入由61亿元增加到142亿元，均比“十五”末翻了一番。

五年项目攻坚，发展后劲明显增强。“十一五”期间，完成全社会固定资产投资5788.7亿元，是“十五”时期的4.3倍。实施千万元以上项目1.1万个、完成投资4059.3亿元；其中亿元以上项目1099个、完成投资1783亿元，10亿元以上项目93个、完成投资576亿元。中硅公司5000吨多晶硅、中信重工新重机工程、洛阳石化油品质量升级改造、中铝洛铜10万吨电子铜板带、香江万基80万吨氧化铝、伊电集团3×30万千瓦机组、华阳孟津电厂1×60万千瓦机组、西霞院14万千瓦水电机组、725所1万吨海绵钛、国机一拖3.3万台大轮拖、尚德公司180兆瓦太阳能电池片等397个亿元以上项目顺利竣工，万基控股集团40万吨高精度铝箔、河南煤化集团20万吨乙二醇、偃师玄奘故里开发等435个亿元以上项目顺利推进。建成了17个省级产业集聚区，数量居全省第一，累计完成投资1052亿元，实现营业收入4685亿元。西霞院水库、禹门河反调节水库、小浪底南岸灌区孟西总干渠等65项农业基础设施重点工程建成投用，新增有效灌溉面积35万亩、节水灌溉面积13万亩。

五年结构调整，产业优势日益凸显。优势工业实现了由基础原材料行业向五大支柱产业的升级换代。装备制造业方面，一拖集团的大轮拖、中信重工的重型矿山装备市场占有率均居全国首位；电力产业总装机容量达到909.9万千瓦、占全省的五分之一；石化产业已形成1000万吨炼油、21.5万吨PX、32.5万吨PTA、40万吨聚酯、36万吨涤纶长短丝产能；硅光电产业已形成从硅材料到光伏电池组件的完整产业链，年销售收入占全省的65%，多晶硅产能占全国的10%，光伏电池片及组件产能居国内前列；新材料产业已形成125万吨电解铝、80万吨氧化铝和38万吨铝深加工产能，中钢洛耐建成了规模世界第一的碳化硅制品基地，725所钛板带产能居全国前列。战略新兴产业从无到有、不断壮大。新能源领域已形成1.8亿安时锂离子动力电池、600套风电叶片产能；节能环保装备领域，中信重工纯低温余热发电设备和新型干法水泥生产线市场占有率分别达到50%和80%，北玻公司低辐射镀膜玻璃设备市场占有率超90%；生物制药领域，普莱柯生物的猪病毒疫苗市场占有率超过80%。我市先后荣膺中国制造业名城、国家新材料高技术产业基地、硅材料及光伏高新技术产业化基地和节能环保装备国家新型工业化产业示范基地等荣誉称号。现代服务业实现了提质升级。文化旅游业支撑作用明显增强，洛阳片区大遗址保护工程被列为国家大遗址保护重大项目，隋唐城、汉魏故城、偃师商城等保护展示工程加快实施，定鼎门遗址博物馆、匾额博物馆、河南壁画馆等7 个新博物馆建成开放。全市 5A 级景区达到2家、 4A 级13家。河洛大鼓等7个项目入选国家非物质文化遗产名录。全省金融副中心地位不断巩固，中信、光大、兴业、民生、浦东、招商银行，齐鲁、中原证券以及安诚、民安财险等一大批金融、证券、保险机构相继入驻，全市金融机构达66家。信息产业异军突起，太平洋门户网站和金蝶、用友软件等知名信息企业落户洛阳，恒生科技园、炎黄科技园等十大重点信息产业项目加快建设，中移动2万座席客服中心等项目推进顺利。房地产完成投资617亿元，竣工商品房2153万平方米，分别比“十五”期间增长3.8倍和1.5倍。

五年拓展提升，城市建设突飞猛进。高标准规划建设城市，五年累计完成投资952亿元，市区规划面积由“十五”末的544平方公里扩大到694平方公里，建成区面积由121平方公里扩展到170平方公里，实现了一跨洛河、再跨伊河发展。城市功能更加完善。新区核心区初具规模，体育中心、歌剧院、博物馆、高铁龙门站等一大批标志性建筑竣工投用，一座高楼林立、环境优美的现代化新城区屹立于洛水之畔。新区伊滨区扬帆启航，职教园区、撤村并城小区建设快速推进。洛河以北城区改造成效显著，定鼎北路、凯旋西路、九都西路等31条主次干道建成通车，改造背街小巷386条，市区道路由 582公里 增加到 702公里 。新建了凌波大桥、瀛洲大桥、新西苑桥、新洛阳桥、李楼特大桥，形成了七桥飞架洛河的交通格局，拉大了城市框架，畅通了南北交通。高速公路、干线公路通车总里程分别达到 246.5公里 、 1953.6公里 。洛阳机场改扩建工程竣工投用，新增航线4条。城市环境更加宜居。大力实施碧水蓝天青山绿地工程，形成了“林在城中绕、城在山水间”的城市景观。森林覆盖率达到45%，绿地面积比“十五”末增加15%。全市空气质量优良天数连续五年300天以上。城市形象更加靓丽。经过全市上下艰苦努力，我市被命名为国家卫生城市。成功举办2009世界邮展，成为我国继北京之后的第二个举办城市。大遗址保护高峰论坛、秦汉－罗马四大文明展、省第十一届运动会圆满成功，洛阳牡丹花会升格为文化部与河南省政府联办的中国洛阳牡丹文化节。我市先后荣膺中国十大最佳魅力城市、全国节能减排二十佳城市、全国双拥模范城五连冠、全国人防先进城市、全国金融生态示范市、倾国倾城·最值得向世界介绍的中国名城、感动世界的中国品牌城市、世界特色魅力城市200强、全球网民推荐的中国十大旅游城市、最具海外影响力的中国城市、中国低碳旅游示范市等荣誉称号。

五年强力突破，发展活力明显提升。自主创新能力持续增强。新增各类研发机构33家；其中国家重点实验室4家，实现了全省零的突破，数量占全省的80%。承担国家“863”计划项目和省重大科技专项29项，总数居全省第一。获得国家科技进步特等奖和一等奖10项，占全省的80%。我市被列为国家创新型试点城市，荣膺全国科技进步先进市、国家知识产权示范市称号。国企战略重组实现重大突破。先后引进了中铝公司、中钢集团、中集集团、国机集团、中色总院、中船重工、中建材、河南煤化集团等40多家优强企业，引进资金210亿元，盘活资产144亿元，一大批老企业重新焕发了生机。对外开放水平不断提高。五年实际利用外资38.7亿美元、市外境内资金1082.7亿元，分别是“十五”期间的10.7倍和7.4倍。外贸出口是“十五”期间的2.5倍。家乐福、沃尔玛、台塑集团、正大集团、恒大地产等一大批国内外知名企业相继入驻洛阳。民营经济发展突飞猛进。规模以上民营工业企业达到1500家，个体工商户22.5万户，分别是“十五”末的1.8倍和2.2倍；民营经济占GDP的比重由42.2%提高到48.4%。县域经济支撑作用不断增强。9县（市）的生产总值占全市的比重达到59.7%，财政一般预算收入占全市的比重达到44.7%。

五年倾力民生，人民群众得到更多实惠。“十一五”期间，全市各级财政用于民生的支出累计达到525亿元。教育事业蓬勃发展。新建、改造学校435所，中小学校D级危房全部消除。累计落实“两免一补”和助学金15.5亿元，惠及学生400万人次。我市顺利通过国家“两基”验收。高中阶段毛入学率达到90%，普通高招本科上线率由“十五”末的20%提高到37%。成功组建了洛阳理工学院。卫生服务体系更加健全。在全省率先建立了城镇职工基本医疗保险、城镇居民基本医疗保险、新型农村合作医疗、城乡困难群众补充医疗保险等覆盖城乡、互为补充的医疗保险体系。实施优势医院倍增计划，新建、改扩建县级医院21所、乡镇卫生院和社区卫生服务中心172所、标准化村卫生室2982所。社会保障体系不断完善。五年累计新增就业再就业74.3万人；发放小额贷款17.5亿元、居全省第一，支持群众创业就业15.8万人。在全省率先实行社会保险“五险合一”。农村养老保险参保人数达到65.7万人。各项社会保险参保人数比“十五”末增加256.8万人次。城乡低保和五保对象达到40万人，基本实现了应保尽保。困难群众住房问题得到有效解决。建成经济适用房134.9万平方米、廉租房76.7万平方米，改造大企业职工住房96.7万平方米，5.2万户困难群众喜迁新居。在全省率先完成农村困难群众危房改造。农村生产生活条件明显改善。搬迁扶贫1.67万户、整村推进608个村，农村贫困人口减少30万人。解决安全饮水110万人。县乡公路、通村道路通车里程分别达到3726.8公里和11821.3公里。全市基本实现了行政村通电话、通客车、通水泥路、通广播电视。2010年，城镇居民人均可支配收入和农民人均纯收入分别比“十五”末增加7919元和2777元。

五年攻坚，五年跨越。回顾五年来的工作，发展的历程极不平凡，取得的成绩实属不易，积累的经验弥足珍贵。一是一心一意谋发展。始终坚持发展是硬道理，牢固树立加快发展、科学发展的意识，抢抓机遇，注重运作，既抓当前，又抓长远，发展的基础更加牢固。二是突出重点抓特色。进一步理清工作思路，突出重点，培育亮点，靠工业增强实力，靠城市提升形象，靠文化旅游彰显特色，靠农业稳固基础，靠调整促进转型，靠科技提升竞争力，发展的质量不断提升。三是统筹兼顾促协调。既重视经济建设，又重视生态建设和社会建设；既抓好中心城市发展，又注重城乡一体发展；既注重扩总量，又注重调结构，发展的协调性进一步增强。四是改革创新破难题。针对经济社会发展中的难点问题，大胆改革，善于创新，形成了比较完善的政策体系、工作机制和工作措施，发展的能力进一步提高。五是千方百计惠民生。始终坚持以人为本，执政为民，发展的成果更多地惠及人民群众。六是精诚团结重实干。全市上下心往一处想，劲往一处使，干事创业的氛围日益浓厚，发展的合力进一步增强。这些经验，必须一以贯之地加以持续，加以深化，加以提升。

各位代表！五年来我们取得的成绩，是省委、省政府和市委正确领导的结果，是市人大、市政协监督支持的结果，更是662万洛阳人民团结拼搏的结果。在此，我代表市人民政府，向辛勤工作在各条战线的全市人民，向人大代表、政协委员，向各民主党派、人民团体，向驻洛部队、武警官兵和公安干警，向离退休老领导、老同志，向所有关心支持洛阳发展的社会各界人士，表示衷心的感谢并致以崇高的敬意！

成绩固然可喜，但发展中的问题更须理性应对。经济结构不尽合理。第三产业总量偏小，第二产业层次有待提高，高新技术产业占比偏低。发展方式依然粗放。发展的质量和效益还不够高，科技优势尚未充分发挥，节能减排压力依然较大，安全生产形势还比较严峻。改革改制有待深化。企事业单位运行机制不够优化，中小企业改制遗留问题尚未得到彻底解决，发展活力还没有充分释放。民生建设仍需加强。农村农业基础依然薄弱，扶贫攻坚任务仍很艰巨，教育、医疗、就业、住房等关系群众切身利益的问题尚未得到妥善解决。发展环境尚待优化。政府部门作风有待转变，办事效率有待提高。以上这些问题，我们一定在今后工作中高度重视，采取有效措施认真加以解决。同时，诚挚欢迎各位人大代表、政协委员和社会各界对政府工作多提宝贵意见。

二、继往开来，“十二五”我们肩负着福民强市的发展重任

“十二五”时期是洛阳全面建设小康社会、走在中原崛起前列的关键期，更是洛阳大有作为的重要战略机遇期。面对中央、省“十二五”发展的新要求，面对中原经济区上升至国家战略的新机遇，面对全市人民过上更加美好生活的新期盼，市委九届十六次全会集中各方智慧，科学谋划、确定了“十二五”洛阳经济社会发展的总体目标、总体布局、总体定位、基本路径和基本保障，描绘了“十二五”发展的宏伟蓝图。这个战略部署顺应发展潮流，切合洛阳实际，符合人民意愿，是我市全面建设小康社会的行动指南。我们要紧紧围绕“福民强市”的总体目标，按照中原经济区建设的总体要求和“一中心五组团四支撑”的总体布局，依托产业兴市、创新活市、精神立市、依法治市四条基本路径，强化福民强市考评机制的基本保障，推动新型工业化、特色城镇化和农业现代化协调发展，形成中原经济区“三化”协调发展的洛阳示范板块；依托较为雄厚的工业基础，靠全面创新、产业升级实现老工业基地振兴，建成特色明显的新型工业化城市；依托十三朝古都、河洛文化源头的优势，靠河洛文化传承打造国际文化旅游名城；依托秀美的自然风光，靠环境保护和生态建设打造生态良好的宜居山水城市。

——总量再翻一番。全市生产总值年均增长12%，高于全国和全省水平，到2015年，达到4650亿元，在经济总量上“再造一个新洛阳”，使洛阳在全国大中城市的排名明显提升；地方财政一般预算收入达到284亿元，比“十一五”末翻一番。

——结构更加优化。农业产业化、优质化、机械化水平明显提高。装备制造等五大支柱产业主营业务收入达到7000亿元左右，建成在国内外具有重要影响的五大产业基地，从“洛阳制造”走向“洛阳创造”；新能源、电子信息等五大新兴产业的主营业务收入突破1000亿元。到2015年，服务业增加值占GDP的比重提高到37%，把洛阳打造成为国际文化旅游名城和国际重要旅游目的地、省域金融副中心、中西部重要的物流节点和信息服务外包基地。

——城乡更趋协调。“一中心五组团四支撑”的城镇化总体布局基本成型，城镇化率达到52%。城市建成区面积进一步扩大，在空间上“再造一个新洛阳”。国家环保模范城市和全国文明城市创建成功，洛阳在国内外的知名度、认可度和美誉度大幅提高，成为宜游、宜居、宜业的山水园林和生态休闲名城。

——活力更加充沛。重要领域和关键环节的改革取得重大突破，制约发展的瓶颈因素得到有效破解；开放水平进一步提高，利用外资和进出口总额实现高质量翻番；科技对经济增长的贡献率达到65%；全民创业氛围更加浓厚，民营经济成为经济发展的主力军；政府效能进一步提升，发展环境进一步优化。

——人民更加幸福。城镇居民人均可支配收入、农民人均纯收入超过全国平均水平。上学难、看病难等问题得到有效缓解，低收入群体住房保障问题明显改善，广覆盖、保基本、多层次、可持续的社会保障体系更加完善。社会和谐程度和文明程度进一步提高，人民群众安全感和幸福指数明显提升。

《洛阳市国民经济和社会发展第十二个五年规划纲要（草案）》及说明已印发大会，请一并审议。

三、开拓进取，2011年我们要努力为“十二五”开好局、起好步

2011年是 “十二五”规划的开局之年。今年政府工作的指导思想是：深入贯彻党的十七大、十七届五中全会和中央、省委经济工作会议精神，以科学发展为主题，以加快转变发展方式为主线，以福民强市为目标，突出“一抓三创”工作重点，坚决打好项目建设、经济转型、机制转换、城市提升、民生改善、环境创优“六场攻坚战”，实现经济、社会、生态建设主要指标高于全省平均水平，城乡居民收入的增加、幸福指数的提升和对各级干部的满意度高于全省平均水平，确保社会稳定大局绝对不出大的问题，确保安全生产绝对不出大的问题，努力在中原经济区建设中当示范、做标兵、上水平。

建议2011年全市经济社会发展的主要预期目标是：地区生产总值增长12%；地方财政一般预算收入增长13%以上；全社会固定资产投资增长21%，其中城镇投资增长21%；社会消费品零售总额增长17%；外贸出口、实际利用外资完成省定目标；居民消费价格涨幅控制在4%左右；城镇居民人均可支配收入增长9%，农民人均纯收入增长9%；单位生产总值能耗降低3.5%；人口自然增长率控制在6.5‰以下；新增城镇就业10万人以上，城镇登记失业率控制在4.5%以内。

完成上述目标任务，必须坚持六个“更加突出”。一是更加突出科学发展这个主题，坚持在发展中调整、在发展中转变、在发展中提高。二是更加突出项目建设这个抓手，创新思路引项目、着眼长远谋项目、坚定不移上项目，做大经济总量，促进产业升级。三是更加突出扩大投资这个关键，千方百计扩大投资规模，增加投资强度，优化投资结构，为长远发展奠定坚实基础。四是更加突出改革改制这个动力，以更大的决心和勇气，着力破除体制机制障碍，进一步激发发展的内在活力和内生动力。五是更加突出民生这个根本，不断加大民生投入，着力解决好人民群众最关心、最直接、最现实的问题，让人民群众共享发展成果。六是更加突出发展环境这个保障，下大力提升行政效能，提高服务质量，让企业满意、让投资者满意、让群众满意。

重点做好九个方面的工作。

（一）扩大总量，优化结构，不断提高工业发展水平。坚持一手抓总量扩张和速度提升，一手抓存量调整和增量优化，推动工业结构转型升级。以项目建设攻坚战为载体，以十大工业结构调整重大示范项目为龙头，全年计划实施千万元以上工业结构调整项目535个、完成投资730亿元；其中新开工项目286个、完成投资397亿元，竣工项目351个、完成投资349亿元。一是在经济运行协调上下功夫。完善市场开拓、融资服务、困难企业帮扶等服务企业长效机制。加强对重点产业、重点企业的分析、监测，及时解决倾向性、苗头性的问题。完善电煤等要素保障机制。抓好已竣工项目的达产达效，尽快形成生产效益。加快企业上市步伐，力争北玻公司、通达电缆、隆华传热、普莱柯生物、中信重工IPO上市，推动洛拖、洛钼回归A股。二是在做大做强五大支柱产业上下功夫。装备制造业要开工建设中信重工高端装备制造基地、国机重工产业园等102个项目；加快南车集团洛阳机车城轨装备基地、中信重工电液控制装备制造等132个在建项目建设；确保LYC轴承公司老厂区整体技改、轴研科技精密轴承等78个项目年底前竣工投产；积极推进国机一拖10万辆重卡、恩梯恩LYC公司5000万套汽车轴承等项目的前期工作。新材料产业要加快万基控股集团40万吨铝板带箔、20万吨钛白粉等53个在建项目建设；确保伊电集团25万吨高精度铝板带、龙鼎铝业60万吨铝箔一期、725所5000吨钛板带及换热器等66个项目年底前竣工投产；争取黎明化工院电子气体等62个项目尽快开工；积极推进725所科技产业园、双瑞万基钛业1万吨海绵钛及钛材料等项目前期工作。硅光电产业要加快中硅公司副产物循环利用、超日光伏产业园25兆瓦切片及100兆瓦太阳能电池片扩建等13个在建项目建设；确保赛阳硅业100兆瓦太阳能电池组件、四季沐歌太阳能基地二期等12个项目竣工投产；争取中硅公司120兆瓦太阳能电池片及组件、中阳光伏公司500兆瓦太阳能电池片等17个项目尽快开工建设；积极推进中硅光伏园、尚德300兆瓦太阳能电池等项目的前期工作。能源电力产业要确保上半年华阳孟津电厂第二台机组建成发电；加快洛阳瑞阳2×30万千瓦机组、义煤集团孟津煤矿等7个在建项目建设；积极推进洛阳核电、洛宁大鱼沟电站、大唐热电2×60万千瓦热电联产等项目的前期工作。石化产业要加快洛阳石化14万吨聚丙烯、河南煤化集团20万吨乙二醇等16个在建项目建设；确保矿业集团氟化氢二期等26个项目竣工投产；争取洛阳石化45万吨PX、河南煤化集团90万吨PTA等25个项目开工建设；积极推进洛阳石化新增1000万吨炼油等项目的前期工作。三是在壮大五大战略新兴产业上下功夫。围绕新能源、电子信息、生物医药、电动汽车、节能环保装备等领域，加快培育骨干企业。加快中航锂电20亿安时锂离子动力电池、中航光电器件产业化基地等项目建设。积极推进中集凌宇电动汽车、中信重工水泥回转窑消纳城市生活垃圾示范工程等项目的前期工作。四是在提高产业集聚水平上下功夫。全市17家产业集聚区计划完成投资650亿元，其中工业投资440亿元；实现企业总收入3100亿元；新开工千万元以上工业项目300个，新入驻企业400家。年底前实现基础设施全覆盖。五是在建设创新型试点城市上下功夫。以建设国家创新型试点城市为载体，实施好创新主体培育等工程和30项重大科技创新项目，开工建设投资5亿元的“863”软件孵化器，新建企业研发中心20家。力争建成1—2个省级创新型产业集聚区，新建一个国家级特色产业基地，努力把高新区建设成国家创新型园区。全年实现高新技术产业总产值600亿元、增加值160亿元。专利申请突破3000件。六是在节能减排上下功夫。加快淘汰落后产能，围绕火电、建材、化工等高耗能、高排放行业，重点抓好24个节能减排项目。大力实施碧水蓝天工程。积极建设循环经济试点市。

（二）旅游带动，集约发展，迅速增加服务业总量。坚持以文化旅游业为重点，以十大服务业重大示范项目为带动，不断拓展新领域，提升服务业发展水平。全年计划实施千万元以上服务业项目136个，总投资368.6亿元，完成投资141.1亿元。一是做大做强文化旅游业。科学编制《国际文化旅游名城规划纲要》，积极申报全国文物旅游示范区。办好中国洛阳牡丹文化节、河洛文化旅游节、小浪底观瀑节和伏牛山滑雪节等，不断提高节会水平。加快龙门、小浪底西霞院、隋唐遗址、汉魏故城等文化旅游产业园区和栾川等省级旅游产业集聚区建设，建设契约博物馆和曹休大将军墓博物馆。继续推进机场建设，以包机、途经等形式开辟国际、国内航线，力争全年机场客运达到40万人次、国际口岸地位得到巩固。加强区域旅游联合，打造历史文化旅游产业带、生态休闲度假旅游产业带和伏牛山旅游产业带。全年接待游客6630万人次，其中入境游51.75万人次；旅游总收入348亿元。二是培育壮大现代服务业。加快中移动客服中心、恒生科技园、IDC及灾备中心等信息服务业项目，厦门合一集团中部（洛宁）市场集群基地、通和农副产品物流园区、国药豫西医药物流中心等项目建设。积极引进保险、证券、期货等金融机构。加快发展研发服务、创业服务、知识产权交易、科技成果转化等科技服务业，抓好高新区专利产品公共服务平台、洛龙炎黄科技园、轴承产业服务平台等项目建设。三是加快提升传统服务业。完善基础设施和公共服务平台，吸引大型服务企业在洛设立总部、营运管理中心、分支机构。加快城乡商贸网络建设，重点抓好正大城市广场、名门广场、升龙广场、宝龙城市广场二期、泉舜财富中心和购物中心等26个大型商业项目，力争完成投资10亿元、建筑面积25万平方米。大力发展家庭服务、养老服务、社区卫生服务等行业。四是引导房地产业健康发展。吸引社会资金参与房地产开发经营。抓住房地产开发商向二、三线城市转移的机遇，引进一批信誉好、实力强的房地产企业，参与商品住宅、产业地产和保障性住房开发。

（三）完善功能，彰显特色，进一步增强中心城市辐射带动能力。以十大基础设施重大示范项目为龙头，打好城市提升攻坚战，努力建设“文化为魂、水系为韵、牡丹为媒、产城融合、带状发展、生态宜居”的魅力洛阳。一是以战略眼光规划好城市。科学编制各类专项规划和控制性详规，争取第四期城市总体规划和土地利用总体规划尽快获批。积极谋划一批体量大、带动力强、对全局发展有战略影响的重大基础项目，进一步提升城市品位。二是以精品理念建设好城市。新区开发计划实施重大项目299个，完成投资300亿元以上。其中新开工项目160个，完成投资182.5亿元。核心区要立足完善功能、优化布局、提升品位，高标准建设高铁龙门站周边、隋唐遗址等重点区域。伊滨区要重点加快路网建设，基本建成水电气暖、污水处理等基础设施，伊滨公园、中央商务区、行政副中心、中央轴带、撤村并城小区、职教园区等重点区域和重点项目要有明显的形象进度。洛河以北城区要启动快速路、环城路建设，打通断头路，改造道路及背街小巷42条。加快老旧居民小区综合整治，改善群众居住环境。加快旧城和城中村改造，全年完成投资170亿元，确保完成拆迁400万平方米，改造城中村21个、开工350万平方米、竣工260万平方米。交通方面重点抓好27个总投资346.5亿元的重大项目，全年完成投资127.6亿元。加快连霍高速洛阳至三门峡段建设，争取连霍高速郑州至洛阳段年底前建成通车；洛栾高速、郑卢高速洛阳段10月底前要完成路基工程。加快武西高速洛阳段等项目前期工作，争取年内开工建设。加快洛偃、洛吉、洛宜等快速通道建设。积极推进洛伊、洛新等快速通道前期工作。抓紧编制市区轨道交通项目规划，力争新郑机场至登封至洛阳轨道交通项目早日开工。三是以特色理念塑造好城市。要做好“文化为魂”这篇文章。加大古城风貌保护力度，尽快开工老城区历史街区保护和整治、老子及道教文化产业园等工程。尽快制订汉魏故城、隋唐城等重要遗址保护、展示和利用规划，启动应天门遗址、永宁寺保护展示，白马寺、关林古建筑群综合整治等工程，建成隋唐城明堂、天堂遗址保护展示工程。充分挖掘洛阳历史文化优势，规划建设一批高档次、高品位、特色明显的文化产业园区。大力发展影视、动漫、演艺等文化产业，积极开发、包装一批具有地方特色的演艺产品，努力把资源优势转化为产业优势。要做好“水系为韵”这篇文章。启动市区水系连通、利用和两岸环境改善等工程，打造连片景观带和休闲带。加快引黄入洛工程建设，年底前完成工程量的70%。抓好洛河生态治理和伊河市区段综合整治。提升改造涧河、氵廛河市区示范段。确保邙山渠综合整治下半年开工建设。要做好“牡丹为媒”这篇文章。扩大市区牡丹种植面积，积极开发牡丹系列产品，拉长产业链条，提升牡丹品牌，彰显花城特色。要做好“生态宜居”这篇文章。积极建设林业生态市，加快城郊生态绿地建设，重点抓好15项市级示范工程，植树造林42.7万亩，森林抚育和改造12.1万亩。四是以现代理念管理好城市。充分利用城市资源，做大做强投融资平台，构建灵活有效的投融资机制。实施城乡面貌三年大提升行动，加快数字化城市管理平台建设，提高精细化管理水平，确保通过国家卫生城市复审。深化“畅通洛阳”活动，采取综合措施，缓解交通拥堵。深入开展创建全国文明城市活动，确保达到创建标准。

同时，加快县城和小城镇建设，抓好偃师、栾川等城乡一体化试点，形成沿陇海、洛湛铁路干线集中布局的城市密集区，辐射带动周边县（市）加快发展。县城建设及改造完成投资96亿元，开工500万平方米。

（四）调整结构，强化基础，全面推进新农村建设。完善强农惠农政策，提升基础保障能力，促进农业增效、农民增收。一是大力调整农业结构。加快孟津、伊川、宜阳、洛宁等粮食核心区建设，确保全市粮食总产稳定在40亿斤以上。加快重点区域蔬菜基地建设，新发展蔬菜4万亩。新增牡丹等花卉林果基地3.5万亩、核桃11万亩。新增奶牛存栏1万头，肉蛋奶总产达到90万吨。二是提升农业产业化水平。确保农业产业化龙头企业达到400家；规范完善农民专业合作社100家，重点扶持培育十佳农民专业合作社。农业产业化经营占农业总产值的比重达到60%。加大培训、信息服务力度，转移农村劳动力135万人，劳务创收85亿元。以龙头企业为载体，按照“依法自愿有偿”的原则，引导农村土地有序流转15万亩以上。三是改善农村生产条件。加快孟西灌区建设，完成病险水库除险加固12座，新增有效灌溉面积3万亩、节水灌溉面积5万亩，治理水土流失200平方公里。加快前坪水库项目前期工作。扶持发展农机专业合作社60家以上。四是加快推进新农村建设。开工新型社区80个，建成50个；硬化新型社区和农村通组通户道路 2000公里 ，新修排水设施 1800公里 ；新建农村社区服务中心100个。发展农村户用沼气1万座，大中型沼气工程30处。解决饮水安全34.6万人。搬迁扶贫2250户1万人，整村推进50个村。

（五）创新体制，转换机制，加快推进各项改革。打好机制转型攻坚战，逐步解决深层次的体制机制问题，为发展注入强大动力。一是不断扩大国企改革改制成果。建立健全现代企业制度，不断提高企业竞争力。鼓励重点骨干企业通过股权并购、股权置换、相互参股等方式重组合作，力争再落地一批大项目、好项目。加快白马集团、人造板厂、煤电集团等企业改革步伐。强力推动市政公用、交通运输、粮食等行业改革。妥善解决好中小企业改革遗留问题。二是确保事业单位改革基本完成。全面加快事业单位改革，确保年底前大头落地。完成医药卫生体制五项改革，政府举办的城市社区卫生服务机构和乡镇卫生院全部实施国家基本药物制度。三是积极推进其它领域改革。深化行政管理体制改革，推进机构编制实名制管理。深化财政金融体制改革。加快文化体制改革，全面完成文艺院团转企改制。稳妥推进集体林权和农电体制改革。

（六）扩大规模，拓宽领域，进一步提升开放水平。坚持把扩大开放作为带动全局的综合性战略举措，以开放促改革、促发展。一是强力推进招商引资行动计划。依托17个产业集聚区，大力吸引沿海地区产业链、产品链的关键环节和龙头企业在洛布点发展，带动企业集群式、板块式转移。舍得拿出优势企业和优质资产引进战略投资者。重点落实好与国机集团、国药集团、恩菲公司、蓝星集团、昊华集团等签订的战略合作框架协议，争取央企加大在洛投资、更多项目落户洛阳。组织好牡丹文化节投资贸易洽谈会等重大招商活动，积极参加厦交会、广交会、高交会、中博会等，力争签约一批大项目、好项目。做好项目策划、包装等基础性工作，有针对性地搞好推介，努力提高招商实效。加大重大招商项目的协调服务力度，着力推进301个亿元以上、总投资1767.3亿元在建项目早日投产，推进261个亿元以上、总投资1249.9亿元签约项目早日开工，促进146个亿元以上、总投资1153.9亿元在谈项目早日签约。二是千方百计扩大出口。全面实施科技兴贸和机电产品出口推进计划，扩大机电产品、高新技术产品出口，稳定劳动密集型产品出口。争取上半年洛阳出口加工区获得国家批准。以产业集聚区、出口加工区等为平台，争取引进一批重量级出口型项目。加快服务外包基地建设，重点培育2个出口超亿美元、20个出口超千万美元的拳头商品。三是大力发展民营经济。坚持市领导重点联系民营企业等制度，及时帮助企业解决发展中的问题。用好3000万元中小企业发展专项资金，对符合国家产业政策、科技含量高、市场前景好、发展潜力大的项目和产品给予支持。积极组织银企对接活动，积极稳妥发展小额贷款公司，支持金融机构建立中小企业信贷“绿色通道”，确保中小企业及民营企业贷款增速高于全部贷款增速。活跃中小企业产权交易市场，开辟多元化融资渠道。选择100家骨干民营企业予以重点倾斜支持，促其做大做强。

（七）着力民生，破解难题，全面提升福民惠民水平。坚持福民优先原则，打好民生改善攻坚战，让人民群众得到更多实惠。一是全力办好福民实事。对照省定、市定十件实事，明确责任，落实资金，强化措施，切实把实事办好、好事办实。市级财政计划用于民生的支出111.7亿元。二是引导支持全民创业。认真落实就业扶持政策，多渠道开发就业岗位，积极扶持自主创业。加强就业技能培训、信息服务和就业援助，重点做好高校毕业生、失地农民、外出务工返乡人员等群体的创业就业工作，新增城镇就业10万人、再就业4万人，引导3万人自主创业。三是完善社会保障体系。扩大养老保险覆盖面，解决好养老保险转移接续和未参保、漏保城镇职工参保问题。完善企业退休人员基本养老金、城乡低保、农村五保供养和优抚对象生活补助自然调整机制。尽快出台与物价指数联动的动态社会救助政策，确保低收入群体的生活不因物价上涨而下降。加强基层医疗卫生体系建设，扩大城镇居民基本医疗保险覆盖面，实施好城乡困难群众补充医疗保险大病医前救助制度。加快保障性住房建设，开工廉租房20万平方米、经济适用房45万平方米，分别竣工25万和30万平方米。开工建设公共租赁住房30万平方米。四是加快发展各项社会事业。积极实施中小学校布局调整，制订县域中小学发展规划，加快学校标准化建设，推进义务教育均衡发展。大力发展学前教育，积极解决“大班额”等突出问题。加快高中向县城集中、初中向乡镇集中，推进完中初高中分离。落实“一保三放开”，鼓励公办学校与社会力量合作办学，构建职业教育多元化办学格局。认真落实教育投入“三个增长”，提高农村义务教育阶段家庭困难寄宿生生活补助标准。确保新区二外秋季建成投用、龙泰中学完成建设任务的70%，启动龙祥中学建设。改扩建城市中小学16所。实施中小学校舍安全工程。抓好市广电文化中心、新区图书馆等公共文化项目建设，建成56个综合乡镇文化站、1500个农家书屋，加快社区文化活动中心、村级文化活动室和广播电视数字化改造等项目建设。加快市民健康信息中心建设。统筹做好人口和计划生育工作，稳定低生育水平。大力支持驻洛部队、民兵预备役和人防建设，争创全国双拥模范城六连冠。

（八）完善机制，夯实基础，确保社会大局稳定和安全生产形势平稳。狠抓社会环境综合治理和安全生产环境综合治理，打好环境创优攻坚战。一是切实维护社会和谐稳定。深入开展社会环境创优活动，坚持和完善领导干部接访等长效机制，建立重大项目、重大决策风险评估制度，强化矛盾纠纷排查，加强人民调解组织建设，认真解决好人民群众反映的问题。加强应急管理，积极预防和妥善处置群体性突发事件和突发公共安全事件。深入开展打黑除恶扫痞专项行动，严厉打击各类违法犯罪活动，提高人民群众的安全感。完善110城市应急平台，提升技防全覆盖实战应用水平。下大力加强重点项目和企业周边治安环境综合治理，为项目建设和企业经营创造良好环境。二是切实加强安全生产工作。深入开展安全环境创优活动，强化源头治理，加强安全生产基础设施建设和培训教育，着力构建安全生产的长效机制。抓好煤矿、非煤矿山、危险化学品等十个重点行业专项整治，积极推进煤矿及非煤矿山井下安全避险等六大系统建设，强力推进尾矿库加固治理，及时消除安全隐患，以铁手腕、硬措施坚决防止重特大事故发生。严格落实安全生产的主体责任和监管责任，加大过程追究、隐患追究、从严追究力度，对失职渎职等行为，要顶格追究、决不姑息。

（九）提升效能，优化环境，全面加强政府自身建设。深入开展 “环境创优年”活动，打好效能提升攻坚战，努力创造全省最优、全国一流的发展环境。增强发展意识，提高发展能力。凡有利于洛阳发展的事，都要紧盯不放、多办快办。注重实干实效，在实干中解决问题，在实干中推动工作，进一步浓厚干事创业、加快发展的良好氛围。增强为民意识，提高服务质量。牢固树立群众利益无小事、企业发展至上的理念，强化公仆意识、服务意识，从群众最不满意的事情抓起，从群众最困难的事情帮起，从群众最期盼的事情做起，倾听群众呼声，关心群众疾苦。坚持一切从群众利益出发，始终站在服务对象的角度想问题、办事情，认真履行首问负责、首席服务官、窗口服务、全程代理等制度，以扎实的工作成效取信于民、造福于民、让人民满意！增强责任意识，提高工作效能。要本着对洛阳人民、洛阳发展负责的精神，多为发展想办法、少为推诿找借口，该办的事情要快办，特殊的事情要特办，外地的经验要参照办。大力倡导雷厉风行的工作作风，科学果断决策，改进运行机制，优化工作流程，减少办事环节，压缩运转时间，不断提高效率。增强法治意识，推进依法行政。严格执行人大及其常委会的决议和决定，自觉接受人大及其常委会的法律监督、工作监督，定期报告重大事项。认真接受人民政协的民主监督，主动听取民主党派、工商联、各人民团体和社会各界人士的意见建议，提高人代会议案、代表建议和政协提案办理质量。重视新闻舆论、群众监督和司法监督，不断提高决策民主化水平。规范行政执法行为，完善执法监督约束机制，确保严格执法、公正执法、文明执法。增强廉政意识，打造廉洁政府。全面落实廉政建设责任制，完善惩治和预防腐败体系，从源头上预防腐败。加强行政监察和审计监督，加大案件查办力度，对各种违法违纪行为严惩不贷。大力倡导艰苦奋斗、勤俭节约的作风，力戒奢侈浪费。加快电子政务建设，扩大政务公开范围，充分保障群众的知情权、参与权和监督权，让权力在阳光下运行。

各位代表！宏伟的蓝图引领我们开拓进取，美好的未来激励我们奋勇拼搏。让我们在省委、省政府和市委的坚强领导下，不负重托，扎实工作，奋力谱写洛阳科学发展、跨越发展、和谐发展的新篇章，为早日实现福民强市目标而努力奋斗！
